# Natural Silica and Plant-Derived Fillers for Dental Composite Resins: A Narrative Review

**DOI:** 10.7759/cureus.94509

**Published:** 2025-10-13

**Authors:** Guanlin Gao, Raja Azman Raja Awang, Wan Muhamad Amir W Ahmad, Noor Huda Ismail

**Affiliations:** 1 School of Dental Sciences, Health Campus, Universiti Sains Malaysia, Kota Bharu, MYS

**Keywords:** dental composite resins, miswak, natural silica, plant-derived fillers, rice husk

## Abstract

The ongoing limitations in antibacterial performance, biocompatibility, and environmental sustainability of traditional dental composite resin fillers have caused urgent issues such as secondary caries and marginal microleakage that require immediate attention. The emergence of natural fillers as sustainable options is significant due to their unique bioactive properties and environmental friendliness.

This review systematically retrieved and analysed relevant literature published between 2015 and June 2025, focusing on the application mechanisms, performance characteristics, and clinical translation potential of natural silica and plant-derived fillers in dental composite resins.

Natural silica derived from agricultural waste, such as rice husks, not only enables high-value utilisation of biomass resources but also improves the mechanical properties of composite resins. Plant-based fillers, like miswak, offer multiple functions, including antibacterial and anti-inflammatory effects, due to their rich bioactive components. However, challenges such as the release behaviour of bioactive components over time, interface bonding stability, and batch consistency remain obstacles to clinical use.

While natural fillers show great promise in dental composite resins, further research is clearly necessary. The potential of surface modification techniques, optimisation of composite formulations, and standardised preparation methods is substantial, and detailed studies can help ensure successful translation from the laboratory to clinical practice.

## Introduction and background

The increasing prevalence of dental caries worldwide, coupled with the persistent challenges of conventional composite resins such as secondary caries formation, insufficient mechanical durability, and the absence of antimicrobial capabilities, underscores the critical need for advanced bioactive restorative materials. Dental composite resins have become the cornerstone material of modern oral restoration since their introduction in the 1960s. Chen established the fundamental compositional framework, including organic resin matrix, inorganic fillers, coupling agents, and initiators, with filler components playing a decisive role in final material performance [1]. However, Ferracane revealed through large-scale clinical follow-up studies the widespread occurrence of problems, including secondary caries, marginal microleakage, and insufficient mechanical strength, severely affecting the long-term success rate of restorations [2]. Zhang et al. demonstrated that the fundamental cause of dental treatment failure lies in biofilm accumulation on material surfaces, whilst traditional materials inherently lack antibacterial capabilities and cannot control microbial infection at the source [3,4].

In recent years, the penetration of green chemistry and sustainable development concepts has brought new perspectives to dental materials research. Pratap et al. explicitly identified the development of novel filler materials with antibacterial properties as an urgent need within the field [5]. Natural-origin filler materials have emerged as frontrunners owing to their unique advantages. Bai et al.'s systematic research confirmed that natural fillers not only possess fundamental advantages of wide availability, low cost, and environmental friendliness, but more importantly, the bioactive components contained in many natural materials can confer multiple functional properties, including antibacterial, anti-inflammatory, and tissue regeneration-promoting effects [6]. Radwanski and colleagues emphasised that the development of bioactive materials aims not only to replace lost dental tissues but also to offer remineralisation and antimicrobial properties [7]. Research by Tzimas et al. revealed an intriguing finding: plant-derived compounds exhibit notable antibacterial activity in the prevention of dental caries, potentially opening a new avenue for dental care. The efficacy of certain extracts was even comparable to that of chlorhexidine [8].

Among numerous natural filler candidates, natural silica and plant-derived fillers have become research hotspots due to their complementary advantages. Noushad et al. first systematically demonstrated that natural silica extracted from agricultural waste such as rice husks can achieve efficient utilisation of biomass resources whilst providing excellent mechanical support for dental composite resins [8]. Complementarily, plant-derived fillers (such as miswak), whilst having relatively limited mechanical properties, add unique functional dimensions to dental materials through their rich bioactive components [9]. Studies conducted by Khairi and other research groups have indicated that silica nanoparticles derived from rice husks can substantially enhance the mechanical properties of dental composite resins [10,11].

Several factors have driven the advancement of biocompatible fillers. Notably, Tanaka et al. discovered that drug-loaded microparticles, made from natural polymers like chitosan, show potent antibacterial properties when integrated into dental composite resins [12]. Importantly, these microparticles maintain the mechanical strength and biocompatibility of the resins, striking a crucial balance between functionality and safety. In a similar vein, Sudhakar and his team found that biopolymers from seaweed boast exceptional biocompatibility, biodegradability, and a range of beneficial activities, including antimicrobial, antioxidant, and anti-inflammatory effects [13,14].

Wang and co-workers highlighted that the use of functional fillers extends beyond enhancing mechanical strength, placing greater emphasis on their bioactive capabilities [15]. Tiskaya et al. showed that materials such as bioactive glass exhibit strong antibacterial properties against oral pathogens [16]. Ahuja and colleagues observed that integrating nanotechnology into dental materials opens new possibilities for enhancing natural fillers [17]. Similarly, Ramesh et al. showed that improvements in surface modification techniques allow for stronger interfacial bonding between natural fillers and resin matrices, leading to better overall material performance [18].

## Review

A comprehensive literature search was conducted to identify relevant studies on natural silica and plant-derived fillers used in dental composite resins. The electronic databases searched included PubMed, Web of Science, and Scopus. The search covered publications from January 2015 to June 2025 to capture recent advances in this field. Search terms included "miswak", "antibacterial", "silica", and "composite".

Inclusion criteria included the following: (1) studies on natural silica or plant-derived fillers in dental composite resins, reporting mechanical properties, antibacterial activities, biocompatibility, or surface modification; (2) original research articles, systematic reviews, and clinical studies (January 2015 to June 2025).

Exclusion criteria included the following: (1) studies on natural materials for non-restorative applications; (2) studies on synthetic fillers only; (3) non-English publications.

**Figure 1:**
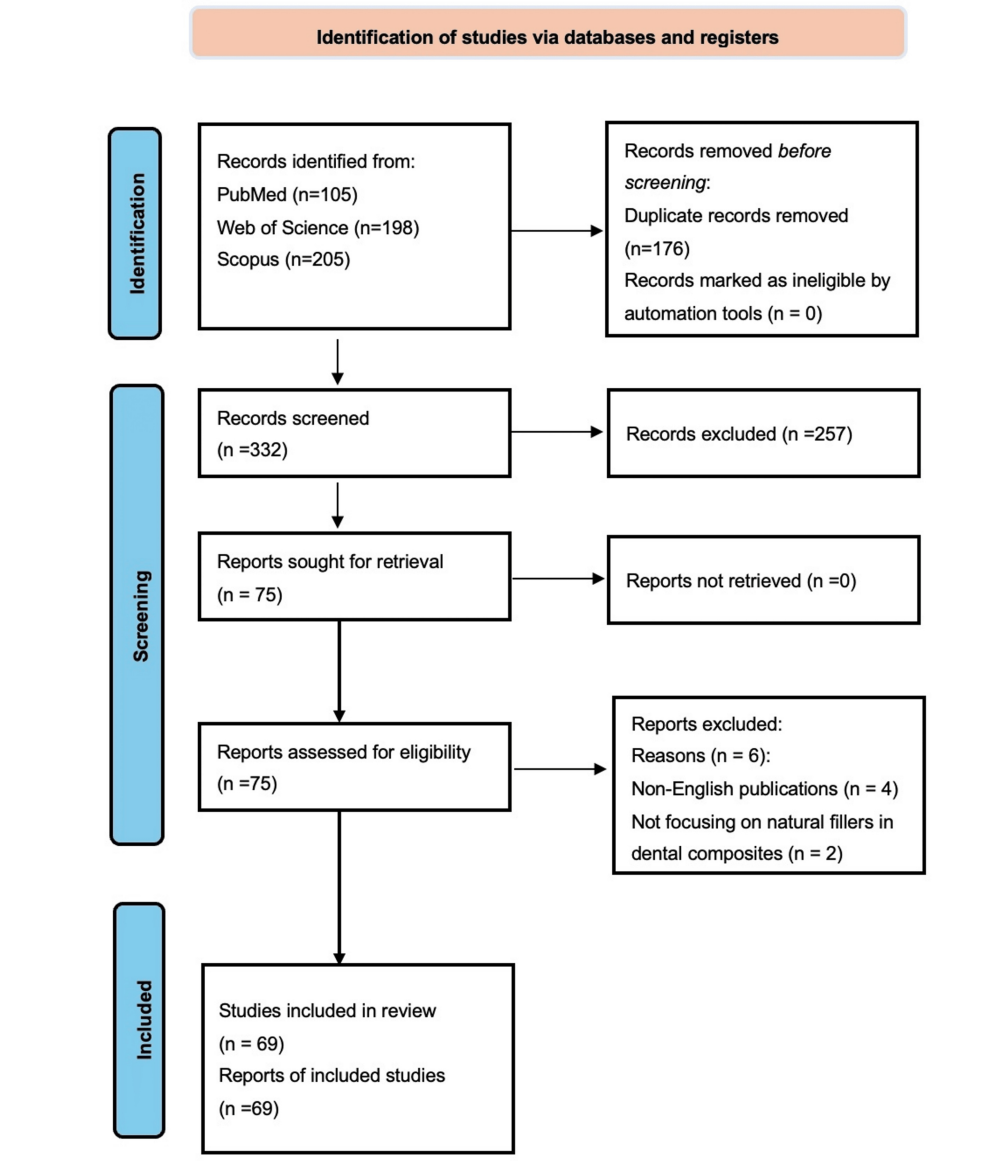
PRISMA flow diagram for the records. PRISMA: Preferred Reporting Items for Systematic Reviews and Meta-Analyses.

A total of 508 articles were initially identified from literature searches across three databases: 105 from PubMed, 205 from Scopus, and 198 from Web of Science. After removing 176 duplicates, 332 articles were screened based on titles and abstracts. Following the initial screening, 257 articles were excluded as they did not meet the pre-specified inclusion criteria. The remaining 75 full-text articles were assessed for eligibility, of which six were excluded due to non-English publications and articles not focusing on natural filler applications in dental composites. Finally, 69 articles met all inclusion criteria and were included in this comprehensive review. Figure 1 illustrates the Preferred Reporting Items for Systematic Reviews and Meta-Analyses (PRISMA) flow diagram of this systematic selection process.

Development background of natural fillers in dental composite resins

Traditional inorganic fillers have gradually exposed inherent limitations through long-term clinical practice. From a functional deficiency perspective, Cheng et al. indicated that traditional fillers cannot provide biological functions such as antibacterial and remineralisation capabilities, leading to susceptibility to secondary caries and gingival inflammation around restorations [19]. From a biosafety perspective, Pratap et al. confirmed that specific synthetic fillers may cause local tissue reactions during long-term use [5]. From environmental sustainability assessment, Shinkai et al. noted that traditional filler production processes commonly involve high energy consumption and pollution [20].

Although traditional fillers perform excellently in mechanical properties, their "mono-functional" design philosophy can no longer meet the diversified needs of modern dental treatment. Existing research predominantly focuses on improving single performance indicators, lacking systematic evaluation of long-term biological effects of materials, and this research perspective limitation constrains overall material performance optimisation.

Natural materials typically possess excellent biocompatibility and can coexist harmoniously with human tissues, as demonstrated by Nordin et al. at the molecular level [21]. Haque et al. showed that many natural materials are rich in bioactive components such as polyphenols, flavonoids, and alkaloids, which can endow materials with multiple functional properties, including antibacterial, antioxidant, and anti-inflammatory effects [22]. Yuan et al. indicated that natural fillers predominantly derive from renewable resources with relatively low production energy consumption, offering significant advantages in environmental friendliness [23]. Carraro et al. confirmed that natural fillers have low raw material costs and relatively simple processing techniques, providing good economic feasibility for large-scale industrial applications [24].

Based on source characteristics and application features, natural fillers can be constructed into a systematic classification system comprising four main categories (Table 1).

**Table 1:** Classification and application characteristics of natural fillers. Table 1 is an original synthesis by the author, compiled from multiple sources and not directly reproduced from any single source.

Filler type	Typical representatives	Main characteristics	Application advantages	References
Mineral-derived fillers	Natural silica, calcium, hydroxyapatite, carbonate	High mechanical strength, good chemical stability	Improve material mechanical properties and structural stability	[25]
Plant-derived fillers	Wood fibres, plant extracts, miswak	Rich in bioactive components	Provide mechanical support and biological functions	[26]
Animal-derived fillers	Collagen, chitosan	Excellent biocompatibility	Promote tissue repair and regeneration	[27]
Microbial-derived fillers	Bacterial cellulose, microbial polysaccharides	Controllable structure, adjustable properties	Precise functional material design	[28]

Natural silica filler applications

Rice husks are a significant by-product of rice processing, with substantial global annual production. Rice husk ash (RHA) contains over 60% silica, offering a rich resource for the large-scale production of natural silica with considerable potential applications [29]. Zulkifli et al.'s research revealed the unique structural advantages of rice husk silica, including porous structure characteristics, large specific surface area, and abundant surface hydroxyl groups, with these structure-function correlations providing scientific explanations for its excellent performance in dental composite resins [30].

Extensive research confirms that silica incorporation can significantly improve comprehensive properties of dental composite resins (Table 2). The addition of 10-15% rice husk silica increases flexural strength by 20-30% through stress dispersion and interface bonding mechanisms [31]. Nanoscale addition improves compressive strength by 7.6% via compressive load stability enhancement. Silanisation treatment increases microhardness by 126.1% through enhanced wear resistance and scratch resistance [32]. Filling levels of 50-60% effectively reduce polymerisation shrinkage by decreasing resin matrix proportion [33]. High hardness dispersion significantly improves wear resistance through hardness characteristics and good dispersion [15].

**Table 2:** Effects of silica on composite resin properties. Table 2 is an original synthesis by the author, compiled from multiple sources and not directly reproduced from any single source.

Performance indicator	Addition amount	Performance enhancement	Mechanism of action	References
Flexural strength	0.1-25.3 wt%	The elastic modulus and shear modulus increased by 47.4% and 21.0%, respectively	Stress dispersion, interface bonding	[31]
Compressive strength	Nanoscale addition	Increase by 7.6%	Compressive load stability	[32]
Microhardness	Silanisation treatment	Increase by 126.1%	Wear resistance, scratch resistance	[32]
Polymerisation shrinkage	50-60% filling	Effective reduction	Reduced resin matrix proportion	[33]
Wear resistance	High hardness dispersion	Significant improvement	Hardness characteristics, good dispersion	[15]

Plant-derived filler applications

Miswak (*Salvadora persica*), as a traditional natural toothbrush plant, has a history of thousands of years of application in the Middle East and Africa. Modern biomedical research has discovered that miswak not only possesses good mechanical properties, but more importantly, its multiple bioactive components make it an extremely promising functional filler in dental composite resins [34].

Miswak's functional properties, derived from its rich chemical composition, are categorised into three main classes (Table 3). Inorganic components, including fluorides, chlorides, silicates, calcium, and phosphorus, promote remineralisation and repair tooth structure [35]. Organic components, including tannins, saponins, flavonoids, alkaloids, and volatile oils, provide antibacterial, anti-inflammatory, and antioxidant functions [36]. Fibre components consisting of natural plant fibres offer mechanical reinforcement and enhance composite resin mechanical properties [37].

**Table 3:** Chemical components and functional properties of miswak. Table 3 is an original synthesis by the author, compiled from multiple sources and not directly reproduced from any single source.

Component category	Main components	Functional properties	Application value	References
Inorganic components	Fluorides, chlorides, silicates, calcium, phosphorus	Promote remineralisation	Repair and strengthen tooth structure	[35]
Organic components	Tannins, saponins, flavonoids, alkaloids, volatile oils	Antibacterial, anti-inflammatory, antioxidant	Natural biological protection functions	[36]
Fibre components	Natural plant fibres	Mechanical reinforcement	Enhance composite resin mechanical properties	[37]

Ayoub et al.'s research revealed the complexity of miswak's antibacterial activity, highlighting not the action of a single component, but synergistic effects of multiple bioactive components through different mechanisms (Table 4). Benzyl isothiocyanate (BITC) represents the primary antibacterial constituent of miswak, effectively eliminating periodontal disease-causing bacteria through membrane potential disruption, thereby considerably diminishing oral pathogen populations [38]. Essential oil components exhibit broad-spectrum antibacterial activity with significant inhibition against *Acinetobacter baumannii* and *Pseudomonas aeruginosa* [39]. *S. persica* aqueous extract encompasses various antioxidant compounds, including lycopene and α-linolenic acid, exhibiting both gastric mucosal protective effects and the capacity to suppress bacterial acidogenesis [40].

**Table 4:** Classification and characteristics of miswak antibacterial mechanisms. Table 4 is an original synthesis by the author, compiled from multiple sources and not directly reproduced from any single source.

Antibacterial component category	Main active components	Antibacterial mechanisms	Clinical significance	References
*S. persica* petroleum ether extract (SPE)	Benzyl isothiocyanate	Penetrates the bacterial outer membrane and disrupts the redox system	Significant inhibitory effects against common oral pathogens	[38]
Essential oil components	Volatile oils, terpenes, aromatic compounds	Broad-spectrum antibacterial activity, significant inhibition against *Acinetobacter baumannii* and *Pseudomonas aeruginosa*	Prevention and control of oral infections, providing a biological protection foundation	[39]
*S. persica* aqueous extract	Lycopene, α-linolenic acid, oleic acid, lycoxanthin, retinoic acid	Inhibit bacterial acid production	Construct preventive functional materials, enhance tooth caries resistance	[40]

Although miswak demonstrates good antibacterial activity, research on the release kinetics of its bioactive components remains insufficient. Most in vitro studies lack simulation of complex oral environments, particularly evaluation of antibacterial effects under conditions involving pH changes and salivary protein interference.

Chaaben et al.'s process research established standardised procedures for miswak filler preparation, including three key steps (Table 5). Raw material pretreatment involves washing, drying, and grinding to remove surface impurities and reduce moisture content [41]. By accurately screening to control the average particle size to be less than 40 μm, it ensures uniform dispersion of the filler and avoids stress concentration [35]. Surface modification using silane coupling agent treatment provides chemical bonding and improves compatibility [42].

**Table 5:** Standardised operation procedures for miswak raw material pretreatment. Table 5 is an original synthesis by the author, compiled from multiple sources and not directly reproduced from any single source.

Process stage	Main operations	Functional characteristics	Application value	References
Raw material pretreatment	Washing, drying, grinding	Remove surface impurities, reduce moisture content	Ensure product quality stability, obtain preliminary particulate materials	[41]
Particle size control	Precise sieving, particle size to be less than 40 μm	Ensure uniform filler dispersion, avoid stress concentration	Provide technical assurance for material performance optimisation	[35]
Surface modification	Silane coupling agent surface treatment	Chemical bonding, improve compatibility	Enhance overall composite material performance and stability	[42]

Al-Bayaty et al.'s systematic research confirmed that composite resins containing miswak fillers exhibit excellent antibacterial performance [43]. Asma et al. demonstrated that miswak fillers can effectively inhibit bacterial biofilm formation [44]. In vitro studies indicate that miswak fillers' antibacterial activity possesses good stability, maintaining practical antibacterial effects [45].

Chaaben et al.'s mechanical property research revealed that the effects of miswak fillers on composite resin mechanical properties exhibit clear dose-dependent characteristics [41]. A 5% addition produces positive reinforcement effects, but performance decreases beyond 10% [41,46]. Wassel et al. elucidated how calcium, phosphorus, fluoride, and other mineral components in miswak synergistically promote tooth remineralisation processes [47]. Upon miswak utilisation, there is a prompt increase in salivary calcium and chloride concentrations, accompanied by a simultaneous reduction in phosphate content and pH levels. However, saliva serves as a reliable source of calcium and phosphate, whilst demonstrating pH buffering properties that assist in preserving supersaturated conditions with respect to dental minerals. This mechanism subsequently provides effective protection against tooth demineralisation under acidic conditions [21].

Composite application of natural fillers

Elfakhri et al. indicated that single fillers cannot simultaneously meet multiple requirements of dental composite resins in mechanical properties, biological functions, and aesthetic effects [48]. Sheng et al. systematically confirmed that the composite application of rice husk silica and miswak can achieve synergistic enhancement by fully exploiting complementary advantages [11]. Crăciunescu et al. elucidated the implications of this synergistic mechanism, ensuring fundamental material reliability whilst achieving effective therapeutic function delivery [49].

Zanchi's research systematically evaluated the silanisation effects of gamma-methacryloxypropyltrimethoxysilane (γ-MPS) at different concentrations, finding that 3% weight percentage achieved the highest flexural strength, whilst 1% concentration showed optimal elastic modulus, confirming that low concentrations (1-3%) are more effective [50]. Research found that rice husk silica content reaches 87-97%. After γ-MPS silanisation treatment, rice husk silica composite materials achieved a flexural strength of 82 MPa and flexural modulus of 6.8 GPa, demonstrating excellent comprehensive performance [51].

Performance evaluation and characterisation

Dental composite resin performance evaluation requires systematic analysis from multiple dimensions (Table 6). A study assessed the Vickers hardness of six resin composite materials under different polymerisation modes [52]. Surface properties evaluation through roughness measurement shows that exceeding the 0.2-0.5 μm range affects aesthetic effects and bacterial adhesion [53]. Rheological properties assessment via shear thinning testing determines operational convenience and clinical operability [54]. Microstructure analysis using scanning electron microscopy/energy dispersive X-ray spectroscopy (SEM/EDX) reveals dispersion characteristics and interface bonding [55]. Evaluation of antibacterial properties through inhibition zone/minimum inhibitory concentration/biofilm testing provides a qualitative and quantitative assessment [56]. Biocompatibility assessment via MTT (3-(4,5-dimethylthiazol-2-yl)-2,5-diphenyltetrazolium bromide)/cell adhesion/genotoxicity testing ensures clinical safety [57].

**Table 6:** Performance evaluation methods for natural filler composite resins. SEM: scanning electron microscopy; EDX: energy dispersive X-ray spectroscopy; MIC: minimum inhibitory concentration; MTT: 3-(4,5-dimethylthiazol-2-yl)-2,5-diphenyltetrazolium bromide. Table 6 is an original synthesis by the author, compiled from multiple sources and not directly reproduced from any single source.

Evaluation dimension	Testing methods	Key indicators	Clinical significance	References
Mechanical properties	Vickers hardness testing	36-105 HV	Wear resistance evaluation	[52]
Surface properties	Roughness measurement	0.2-0.5 μm	Aesthetic effects, bacterial adhesion	[53]
Rheological properties	Shear thinning testing	Operational convenience	Clinical operability	[54]
Microstructure	SEM/EDX analysis	Dispersion, interface bonding	Structure-property relationships	[55]
Antibacterial properties	Inhibition zone/MIC/biofilm	Qualitative and quantitative evaluation	Infection control	[56]
Biocompatibility	MTT/cell adhesion/genotoxicity	Safety assessment	Clinical safety	[57]

Current performance evaluation systems are primarily based on in vitro testing, lacking accurate simulation of complex oral environments. Notably, long-term performance evaluation under multi-factor coupling effects, including dynamic loading, temperature cycling, and pH changes, requires improvement.

Natural filler composite resins face three core challenges limiting clinical application. Time-related issues involve the overly quick release of bioactive components, causing sudden pH shifts and functional loss, as shown by Chatzistavrou et al. [58]. Environmental sensitivity problems occur where oral environmental changes cause filler-matrix interface deterioration, as demonstrated by Hampe et al. [59]. Contradictory issues arise where hydrophilic characteristics of natural fillers cause water absorption and swelling, affecting dimensional stability, as confirmed by Bakdash et al. [60].

Research currently shows that existing studies predominantly focus on short-term performance evaluation, whilst long-term stability assessment remains relatively insufficient, limiting accurate judgment of the actual application effects of natural fillers.

Addressing these challenges, researchers have proposed systematic solution strategies. In terms of material design methodologies, Xu et al. proposed that employing a core-shell structure to control the release rate can achieve a sustained release of bioactive components and enhance functional durability [61]. Zheng et al. investigated the enhancement of stability and antibacterial properties through the addition of fluoride-containing fillers [62]. Multi-filler composite applications confirmed by Naguib et al. achieve performance complementarity and overcome single-component limitations [63].

Natural filler composite resin technology is rapidly advancing to become smarter, more functional, and more suitable for industrial applications. Emerging natural fillers show great promise: nanocellulose offers excellent mechanical properties and good biocompatibility [64]. Graphene-polymer nanocomposites greatly impact dental fillers and their antibacterial uses [65]. By utilising nanoparticle surfaces that enhance osseointegration and employing nanoparticles for enamel remineralisation and antibacterial effects, the research highlights the need for further studies on safety concerns and optimising the integration of nanotechnology in clinical applications [66]. In the pharmaceutical industry, the natural corn polymer, zein, can act as a silane coupling agent to stabilise nanoparticles and preserve their nanoscale properties [67]. Additionally, this additive does not diminish the antibacterial activity of magnesium oxide nanoparticles [68]. Algal fillers rich in polysaccharides and minerals have anti-inflammatory and immunomodulatory functions [69].

## Conclusions

Research on applications of natural silica and plant-derived fillers in dental composite resins has achieved significant progress. Rice husk silica, with its excellent mechanical reinforcement properties, environmental characteristics, and cost-effectiveness, can significantly enhance flexural strength, compressive strength, and microhardness of composite resins after surface modification. Miswak fillers possess unique bioactive characteristics, including antibacterial, anti-inflammatory, remineralisation-promoting properties, and excellent biocompatibility, demonstrating significant efficacy in managing oral microbial environments. Current research still faces key scientific challenges: quantitative description of function-structure relationships, theoretical foundations of multi-component synergistic mechanisms, predictive models for long-term stability, and precise matching strategies for personalised applications require improvement. Research gaps include insufficient long-term biological effect evaluation, a lack of performance stability research under complex environments, and the absence of established standardised quality control systems.

The future development roadmap is clear: short term (one to three years) should improve standardised preparation processes and quality control systems, and deepen mechanistic research on surface modification technologies; medium term (three to five years) should develop innovative responsive composite systems and achieve industrialised production of multifunctional fillers; long term (five to 10 years) should construct personalised application platforms and achieve clinical applications of diagnostic-therapeutic integrated innovative materials. Achieving clinical translation requires a multi-disciplinary collaboration platform construction, integrated cooperation between industry, academia, and research, international collaboration network development, and regulatory policy improvement. Natural filler composite resin technology is developing rapidly and, through systematic scientific research, technological innovation, and industrialisation advancement, promises large-scale clinical applications, making significant contributions to oral medicine development and green, functional development of biomedical materials. The study recommends adopting "staged validation - risk assessment - progressive application" strategies, prioritising validation of technological feasibility in low-risk application scenarios, gradually expanding to complex clinical applications.
